# High-Dimensional Mass Cytometry Analysis of Embryonic Antigens and Their Signaling Pathways in Myeloid Cells from Bone Marrow Aspirates in AML Patients at Diagnosis

**DOI:** 10.3390/cancers15194707

**Published:** 2023-09-25

**Authors:** Carmen-Mariana Aanei, Estelle Devêvre, Adrian Șerban, Emmanuelle Tavernier-Tardy, Denis Guyotat, Lydia Campos Catafal

**Affiliations:** 1 Laboratory of Hematology, University Hospital of Saint-Etienne, 42055 Saint-Etienne, France; adrianserban1994@yahoo.ro (A.Ș.); lydia.campos@chu-st-etienne.fr (L.C.C.); 2 Santé Ingénierie Biologie Saint-Etienne, INSERM SainBiose U1059, 42270 Saint-Priest-en-Jarez, France; 3 Plateau de Cytométrie AniRA, SFR BioSciences (UAR3444-US8), 69367 Lyon, France; estelle.devevre@inserm.fr; 4 Department of Clinical Hematology, University Hospital of Saint-Etienne, 42100 Saint-Etienne, France; emmanuelle.tavernier@chu-st-etienne.fr (E.T.-T.); denis.guyotat@chu-st-etienne.fr (D.G.)

**Keywords:** acute myeloid leukemia, mass cytometry, OCT3/4, Nanog, SOX2, SSEA1, SSEA3, β-catenin, p-p38, STAT3/p-STAT3

## Abstract

**Simple Summary:**

Although a number of important studies have compared the phenotypes of myeloid cells from acute myeloid leukemia (AML) patients to those of their normal counterparts, there is currently no specific surface phenotype that can universally recognize these cells across AML patients. In this study, we used mass cytometry to characterize the expression of five major embryonic antigens (EAs) in different myeloid cell types from both AML bone marrow (BM) aspirates and normal samples. Our aims were to determine if EAs are abnormally expressed in AML cells and if they can help discriminate AML cells from their normal counterparts. The deregulation of EAs may contribute to AML leukemogenesis and the adoption of a “leukemic stem cell” phenotype. In addition, to understand how EAs may contribute to myeloid cell dysfunction in AML, we evaluated several signaling proteins (SPs) involved in major signal transduction pathways.

**Abstract:**

Background: Embryonic antigens (EA) regulate pluripotency, self-renewal, and differentiation in embryonic stem (ES) cells during their development. In adult somatic cells, EA expression is normally inhibited; however, EAs can be re-expressed by cancer cells and are involved in the deregulation of different signaling pathways (SPs). In the context of AML, data concerning the expression of EAs are scarce and contradictory. Methods: We used mass cytometry to explore the expression of EAs and three SPs in myeloid cells from AML patients and normal bone marrow (NBM). Imaging flow cytometry was used for morphological assessment of cells in association with their OCT3/4 expression status (positive vs. negative). Results: An overall reduction in or absence of EA expression was observed in immature myeloid cells from AML patients compared to their normal counterparts. Stage-specific embryonic antigen-3 (SSEA-3) was consistently expressed at low levels in immature myeloid cells, whereas SSEA-1 was overexpressed in hematopoietic stem cells (HSCs) and myeloblasts from AML with monocytic differentiation (AML M4/M5). Therefore, these markers are valuable for distinguishing between normal and abnormal myeloid cells. These preliminary results show that the exploration of myeloid cell intracellular SPs in the setting of AML is very informative. Deregulation of three important leukemogenic SPs was also observed in myeloid cells from AML. Conclusions: Exploring EAs and SPs in myeloid cells from AML patients by mass cytometry may help identify characteristic phenotypes and facilitate AML follow-up.

## 1. Introduction

Embryonic antigens (EAs) maintain pluripotency and self-renewal in embryonic stem (ES) cells during early embryonic development. OCT3/4, SOX2, and Nanog are core transcription factors involved in maintaining ES cell pluripotency and differentiation [1]. OCT3/4 belongs to the POU (Pit-Oct-Unc) family of transcription factors and is a master regulator of gene expression. In particular, it regulates Nanog, which, together with SOX2, maintains ES cell pluripotency [2,3]. In adult somatic cells, expression of EAs is normally inhibited. However, previous reports have shown that EAs are re-expressed by cancer cells. A meta-analysis of 4395 patients across 31 studies published until December 2019 revealed that OCT4 is overexpressed in most solid tumors and directly correlates with poor prognosis [4].

For acute myeloid leukemia (AML), a disease characterized by a differentiation block leading to the accumulation of abnormal immature cells, there are few, contradictory studies pertaining to the expression of EAs. Qinjun et al. showed that OCT4A expression is significantly increased in the bone marrow (BM) nucleated cells of patients with AML [5], while Jing et al. showed that *POU5F1B* (the gene encoding OCT3/4) is frequently underexpressed in BM nucleated cells [6]. Additionally, Picot et al. observed that OCT4 expression was higher in less differentiated leukemias, stage-specific embryonic antigen-1 (SSEA-1) levels were increased in more differentiated AML subtypes, and EA expression was variable between different leukemic cell subsets (CD34^+^CD38^−^ versus CD34^+^CD38^+^) [7]. These findings—particularly, those that relate to the different types of AML included and cell populations analyzed in each study—could explain the differences among these reports. Interestingly, however, underexpression of *POU5F1B* in AML patients is associated with unfavorable clinical variables and a poor prognosis [6,7]. A possible explanation for this observation is that EA deregulation may contribute to AML leukemogenesis and the adoption of a “leukemic stem cell” phenotype.

In this study, we used mass cytometry to compare EA expression in different myeloid cell populations from AML cases and normal settings. The main aim of this work was to investigate whether EAs are abnormally expressed in AML cells and if this aberrant expression could be used to optimally discriminate AML cells from their normal counterparts. In addition, we evaluated several important signaling proteins (SPs) such as β-catenin, phospho-p38 (p-p38), STAT3, and p-STAT3, which are involved in different signal transduction pathways regulated by EAs. Identification of deregulated pathways may help discriminate abnormal cells from their normal counterparts, thereby facilitating AML follow-up and advancing the development of new therapeutic approaches.

## 2. Materials and Methods

### 2.1. Study Design

**Figure 1:**
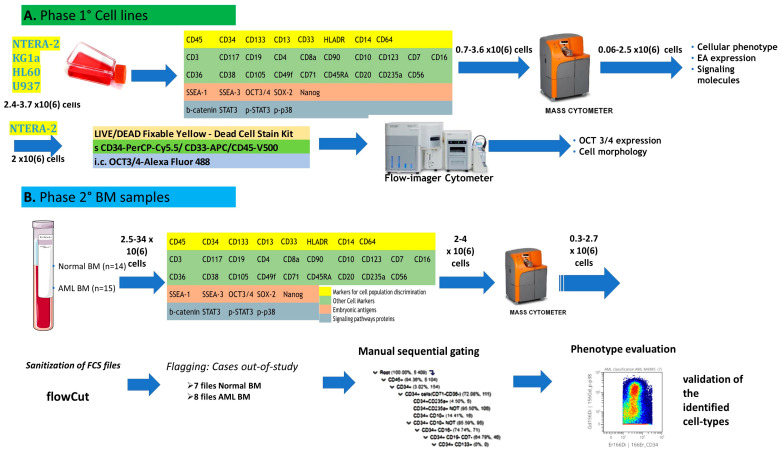
Schematic overview of the study. (**A**) Phase 1: Protocol development. A 36-antibody panel was tested on NTERA-2, KG1a, HL60, and U937 cell lines. Primary antibodies allowing discrimination of cell populations are highlighted in yellow, secondary discriminating antibodies are shown in green, embryonic antigens (EAs) are displayed in salmon, and signaling proteins (SPs) are shown in gray. A total of 2.4–3.7 × 10^6^ cells were stained with the 36 antibodies, 0.7–3.6 × 10^6^ cells per sample were run on the Helios mass cytometer, and data acquired from 0.06–2.5 × 10^6^ cells per sample were used for cellular immunophenotyping and EA and SP expression analysis. Imaging flow cytometry was used for visualization and morphologic characterization of OCT3/4-positive cells. (**B**) Phase 2: Inclusion of patient samples and control subjects. Fourteen normal bone marrow (NBM) aspirates and 15 acute myeloid leukemia (AML) samples were stained with the 36-antibody panel. After data acquisition on the Helios mass cytometer, the resulting FCS files were processed with the flowCut automated data cleaning algorithm. Files containing a reduced number of usable events (<0.2 × 10^6^ events) were removed from the study. Finally, 7 NBM and 8 AML samples were analyzed using a manual sequential method. Analysis of the expression of cell-specific markers was then performed on all identified cell populations to complete their characterization and assign them to a cell type.

The study design is summarized in the flow chart in Figure 1. This study was conducted in two phases. In the first step, the technical protocol was tested on cell lines, whereas the second step included BM samples from newly diagnosed AML patients and normal controls. The cell lines were used to develop our technical mass cytometry protocol, consisting of cell staining, sample preparation, and acquisition. Cellular phenotypes and the expression of EAs and SPs were assessed on cell lines and BM cells from patients and normal controls. Imaging flow cytometry was used for the morphological assessment of OCT3/4-positive compared to OCT3/4-negative cells.

### 2.2. Cell Lines

NTERA-2 human embryonal carcinoma cells (ACC 527) and three leukemic cell lines (KG-1a [ACC 421; human acute myelogenous leukemia; undifferentiated myeloblasts], HL-60 [ACC 3; human promyelocytic leukemia; promyelocytes], and U-937 [ACC 5; human histiocytic lymphoma; pro-monocytic cells]) were obtained from the DSMZ–German Collection of Microorganisms and Cell Cultures GmbH (Leibniz Institute, Braunschweig, Germany). NTERA-2 cells were cultured in Dulbecco’s Modified Eagle’s Medium (DMEM; Sigma-Aldrich, St. Louis, MO, USA) with 10% fetal calf serum (FCS) and 1% penicillin/streptomycin solution (10,000 IU/mL penicillin, 10,000 μg/mL streptomycin). The leukemic cells were cultured in RPMI-1640 with 2 mM L-glutamine, 1% penicillin/streptomycin solution, and 10% FCS at 37 °C in 5% CO_2_.

### 2.3. Patients and Samples

BM samples from 15 AML patients newly diagnosed at the Hematology and Cell Therapy Department of the University Hospital of Saint Etienne were included in this study. Fourteen samples from normal controls (normal bone marrow; NBM) were also included. However, due to sample low quality (samples with poor viability and a high amount of residues due to frequently clogging during sample acquisition), only 7 NBM and 8 AML files contained more than 0.2 × 10^6^ events and were ultimately used for further analysis. The NBM samples used in this study were obtained from healthy BM donors (n = 2) or leftover diagnostic material from non-AML leukemia (n = 2) or multiple myeloma (n = 3) patients who were in long-term complete remission and negative for minimal residual disease. The seven low-quality NBM samples not used in this study came from two healthy BM donors, and from three patients with multiple myeloma in long-term complete remission, one case of follicular lymphoma in long-term complete remission, and a patient with suspicion of idiopathic thrombocytopenic purpura. The healthy donors were aged 22–67 years (median: 51 years), and 45% of patients were females.

AML cases were diagnosed and classified according to the revised 2016 World Health Organization (WHO) criteria [8]. The characteristics of patients included in the final phase of the study are summarized in Table 1.

### 2.4. Mass Cytometry by Time-of-Flight (CyTOF)

Mass cytometry sample preparation procedures are similar to those used for flow cytometry.

NTERA-2 cells: NTERA-2 cells were harvested by trypsinization (Versene–EDTA, Eurobio, France) and leukemic cells by centrifugation (200× *g*, 5 min). Experiments were performed during the exponential growth phase. Approximately 2–4 × 10^6^ cells were fixed and stained with the viability reagent cisplatin and a panel of 36 antibodies (Table S1) in non-autoclaved Eppendorf tubes according to the manufacturer’s recommendations (MaxPar Phospho-Protein Staining Protocol, Fluidigm, San Francisco, CA, USA). Between 0.7 and 3.6 × 10^6^ cells were recovered at the end of the staining procedure, and data were acquired on a CyTOF–Helios mass cytometer (Fluidigm, San Francisco, CA, USA) at the AniRA cytometry facility, SFR BioSciences (UAR3444-US8), Lyon, France.

BM cells: For primary BM samples, lysis of bulk erythrocytes (BD Pharm Lyse buffer, BD Biosciences, Pharmingen, San Diego, CA, USA) was performed to recover the maximum possible number of cells from each sample. A total of 2–4 × 10^6^ cells per sample were then stained with cisplatin in non-autoclaved Eppendorf tubes, fixed, and stained with the antibody panel (Supplementary Table S1), available online. At the end of the procedure, the stained cells were labeled with iridium-containing intercalators and frozen at −80 °C. The sample preparation protocol is detailed in Supplementary Table S2. Samples were shipped on dry ice, and data acquisition on the CyTOF–Helios mass cytometer was performed immediately after sample thawing. Normalization of mass cytometry data was performed with EQ beads (Fluidigm). The average acquisition rate of the Helios mass cytometer was 200 cells/s to prevent clogging. Acquired data were saved as FCS files and exported in a bead-normalized FCS file format ready for downstream analysis.

Data analysis: First, the FCS files were cleaned with the flowCut algorithm, which automatically removes outlier events due to abnormal flow resulting from obstructions and other technical issues. Cases with few evaluable events and those containing only mature cells were excluded from the study. We observed significant cell loss during the staining process; during cell passage through the mass cytometer; during methanol permeabilization, required for phosphoprotein intracellular labeling; and in some cases when significant cell clumping occurred. Ultimately, 7 FCS files for NBM and 8 FCS files for AML samples (5 AML with monocytic differentiation [hereafter called AML M4/M5] and 3 AML without monocytic differentiation [hereafter called non M4/M5-AML]) were used in the final step of data analysis, all of which contained approximately 300,000 events per sample. Data analysis was performed with the OMIQ cytometry analysis platform. First, we attempted to use an automated analysis method based on the Uniform Manifold Approximation and Projection (UMAP) for dimensional reduction algorithm. However, this algorithm failed to appropriately discriminate among cell populations, prompting us to use a classical hierarchical classification method. Two possible reasons may underlie this problem. First, we did not perform barcoding before immunostaining, which would have improved data quality by minimizing the variability between samples. Second, the quality of the primary material was sub-optimal with respect to the initial number of cells, hemodilution, and the presence of serum factors favoring cellular agglomeration. The manual sequential gating strategy (Figure S1) allowed identification of 14 cell populations, including immature myeloid cells such as hematopoietic stem cells (HSCs), CD34^+^ myeloblasts, CD117^+^ myeloblasts, and monoblasts, as well as more mature myeloid cells such as immature monocytic cells, monocytes, and mature neutrophils. The representative dot plots for immunophenotypic characterization of immature myeloid cell populations are displayed in Figure S2. Finally, the phenotype of each identified cell population was fully characterized by evaluating the expression of all 36 antibody-based markers.

Analysis of the frequencies of the populations of interest in our study revealed increased percentages of monoblasts, CD34^+^ myeloblasts, and immature and more mature monocytic cells in AML M4/M5 cases compared to NBM samples. We also observed an increased percentage of CD34^+^ myeloblasts in non-M4/M5 AML samples (Figure S3). This analysis was performed to validate the gating strategy.

### 2.5. Imaging Flow Cytometry

A total of 2 × 10^6^ cells from the NTERA-2 and leukemic cell lines were first stained with the LIVE/DEAD Fixable Yellow Dead Cell Stain Kit (Invitrogen; Thermo Fisher Scientific, Inc., Waltham, MA, USA) according to the supplier’s recommendations. Cells were then incubated with a cocktail of the following surface marker antibodies for 15 min at room temperature, protected from light: PerCP-Cy5.5-labelled mouse anti-human CD34 (clone 8G12, concentration: 5 µg/100 µL, BD Biosciences, San Jose, CA, USA), APC-labelled mouse anti-human CD33 (clone WM53, concentration: 20 µg/100 µL, BD Pharmingen, San Jose, CA, USA), and V-500-labelled mouse anti-human CD45 (clone HI30, concentration: 1 µg/100 µL, BD Horizon, San Jose, CA, USA). Thereafter, cells were permeabilized with BD Perm/Wash Buffer solution (BD Biosciences) and incubated with AlexaFluor 488-labeled mouse anti-human OCT3/4 antibodies (clone 40/Oct-3, concentration: 20 µg/100 µL, BD Biosciences, Pharmingen, San Diego, CA, USA) for 20 min at room temperature in the dark.

Data were acquired using an Attune CytPix instrument (Thermo Fisher Scientific). The compensation settings were obtained with CompBeads (BD Biosciences).

### 2.6. Statistical Methods

Statistical analyses were performed in R studio 4.1.2 (The R Foundation for Statistical Computing platform) using Shapiro–Wilk tests, D’Augustino Pearson tests, Wilcoxon tests, and graphical functions. As the number of samples included in the study was low, intergroup comparisons were performed with the Wilcoxon–Mann–Whitney test.

## 3. Results

### 3.1. Phenotypic and Morphological Characterization of Cell Lines by Mass Cytometry

**Figure 2:**
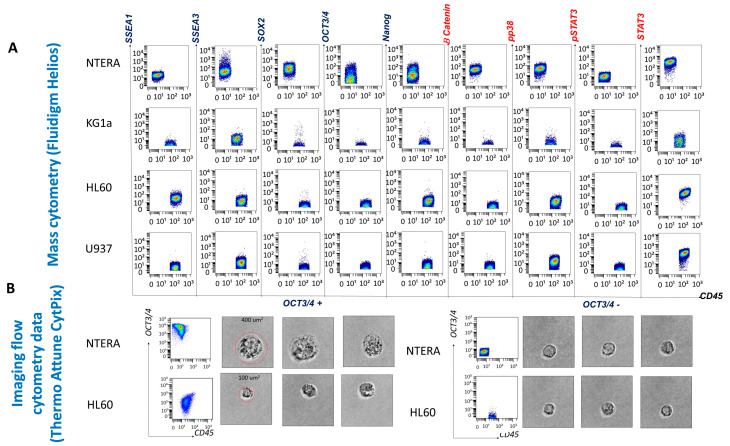
Phenotypic and morphological characterization of the cell lines. (**A**) Evaluation of embryonic antigen (EA) and signaling protein (SP) expression on NTERA-2 pluripotent human embryonal carcinoma cells and three leukemic cell lines (KG1a, HL60, and U937) by mass cytometry. (**B**) Morphological visualization of OCT3/4-positive compared to OCT3/4-negative NTERA-2 and HL60 leukemic cells with imaging cytometry.

Immunophenotyping of the cell lines enabled antibody evaluation and titration, as well as validation of the cell staining and data acquisition protocols. The NTERA-2 cell line, derived from a human embryonal carcinoma, served as a reference for EA expression and allowed us to establish a threshold for positive EA expression (set at > 10^1^). The staining background was evaluated using the NTERA-2 stained with CD45 and was set at 10^1^. Mean metal intensity (MMI) values between 10^1^ and 10^2^ were considered low-intensity expression, and values above 10^2^ were considered high-intensity expression. As we expected, because NTERA-2 is an undifferentiated cell line, it expressed SSEA-3 but not SSEA-1. In addition, NTERA-2 cells expressed low levels of OCT3/4 and Nanog and high levels of SOX2. With respect to the expression of SPs, NTERA-2 expressed low levels of β-catenin and p-p38 and high levels of STAT3. However, only 36.57% of NTERA-2 cells expressed p-STAT3 at low levels (Figure 2).

All three leukemic cell lines expressed low levels of SSEA-3. The promyelocytic cell line HL60 containing more differentiated myeloid cells expressed SSEA-1, while the cell lines KG1a and U937, composed of undifferentiated myeloblasts and promonocytes, respectively, were SSEA-1-negative (Figure 2). SOX2 was not expressed by the leukemic cell lines, whereas only low levels of OCT3/4 and Nanog expression were detected on HL60 cells. The SP STAT-3 was expressed by all leukemic cell lines (with low levels on undifferentiated KG1a myeloblasts and at high intensity on the more differentiated HL60 and U937 cell lines). Overall, HL60 and U937 cells did not constitutively express activated STAT3 (p-STAT3) (2.56% of HL60 cells and 0.68% of U937 cells were p-STAT3 positive). p-p38, a component of the mitogen-activated protein kinase (MAPK) cascade, appeared to be activated only in the more differentiated cell lines (HL60 and U937) (Figure 2). β-catenin was not expressed by any of the leukemic cell lines.

The Attune CytPix is equipped with a bright-field camera that allowed us to investigate the morphology of the OCT3/4-positive cell populations identified by flow cytometry. The NTERA-2 and leukemic HL60 OCT3/4-positive cells were large (approximately 400 µm^2^ for NTERA-2 cells and 100 µm^2^ for HL60 cells), round cells with scant cytoplasm and immature chromatin patterns. Thus, OCT3/4-positive cells morphologically resemble immature blasts.

### 3.2. Expression of EAs and SPs during the Maturation of Normal Myeloid Cells

In our study, mass cytometry facilitated the identification and measurement of EAs and EA-regulated SPs. Therefore, we then explored the expression of EAs and four SPs, β-catenin, p-p38, STAT3, and p-STAT3, in myeloid cells from normal BM samples.

**Figure 3:**
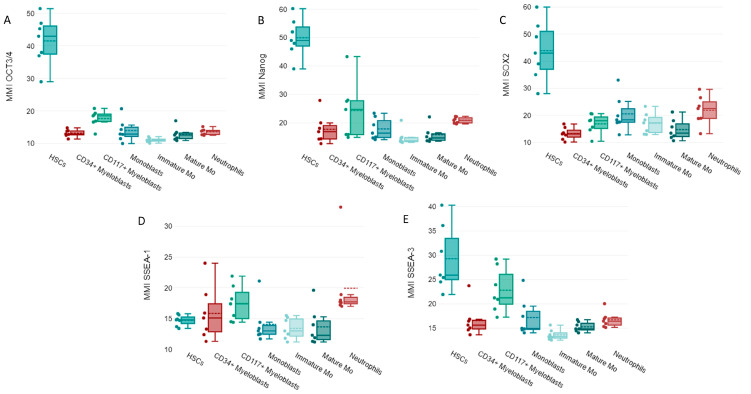
Box plots showing the intensity of expression (MMI) of embryonic antigens (EA): (**A**) OCT3/4, (**B**) Nanog, (**C**) SOX2, (**D**) SSEA-1, and (**E**) SSEA-3 in normal myeloid cells: hematopoietic stem cells (HSCs), CD34^+^ myeloblasts, CD117^+^ myeloblasts, monoblasts, monocytic cells immature (MO), mature MO cells, and neutrophils from seven normal bone marrow samples. The median of the seven MMI values is indicated by the solid lines inside the boxes, the dashed line indicates the mean values, q3 is the upper limit, q1 is the lower limit, and the ends of the whiskers indicate the maximum and minimum values. Individual MMI values are indicated by dots.

In normal samples, high OCT3/4, Nanog, and SOX2 expression levels were observed in HSCs (Figure 3A–C).

The more differentiated cells had reduced OCT3/4, Nanog, and SOX2 expression (Figure 3A–C).

Unlike other EAs, a reduced expression of SSEA-1 was detected in immature myeloid cells from normal bone marrows including HSCs. A relative increased expression of SSEA-1 was observed in normal neutrophils (Figure 3D). Increased expression of SSEA-3 was noticed in normal immature myeloid cells, such as CD117^+^ myeloblasts and monoblasts along with HSCs, and a downregulation of SSEA-3 was noted in mature myeloid cells like monocytic cells and neutrophils (Figure 3E).

**Figure 4:**
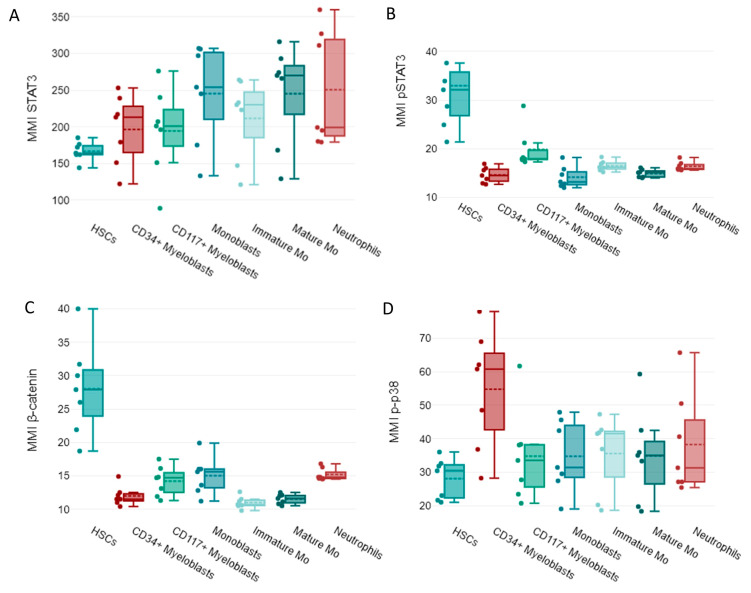
Box plots showing the intensity of expression (MMI) of signaling proteins (SP): (**A**) STAT3, (**B**) p-STAT3, (**C**) β-catenin, and (**D**) p-p38 in normal myeloid cells: hematopoietic stem cells (HSCs), CD34^+^ myeloblasts, CD117^+^ myeloblasts, monoblasts, monocytic cells immature (MO), mature MO cells, and neutrophils from seven normal bone marrow samples. The median of the seven MMI values is indicated by the solid lines inside the boxes, the dashed line indicates the mean values, q3 is the upper limit, q1 is the lower limit, and the ends of the whiskers indicate the maximum and minimum values. Individual MMI values are indicated by dots.

Regarding SP expression, increased levels of activated STAT3 (p-STAT3) were observed in HSCs, whereas un-phosphorylated STAT3 was detected in more mature cells (Figure 4A,B). β-catenin was highly expressed by HSCs, while p-p38 was highly expressed by CD34^+^ myeloblasts in NBM samples (Figure 4C,D).

### 3.3. Evaluation of EA Expression in Immature Myeloid Cells from AML and Normal Samples

**Figure 5:**
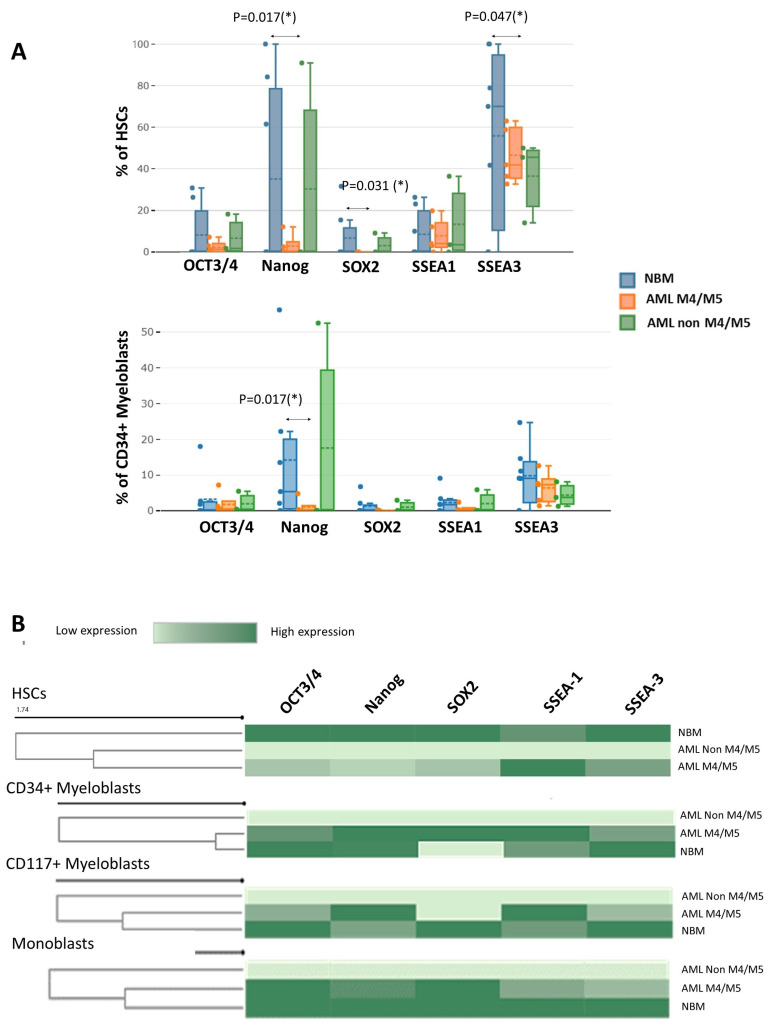
Evaluation of differences in EA expression between acute myeloid leukemia (AML) and normal bone marrow (NBM) by mass cytometry. (**A**) Box plots showing the percentages of EA-positive hematopoietic stem cells (HSCs) and CD34^+^ myeloblasts in AML M4/M5 and non-M4/M5 AML compared to normal controls. The median value is indicated by the solid line inside the box, the dashed line indicates the mean value, q3 is the upper boundary, q1 is the lower boundary, and the ends of the whiskers indicate the maximum and minimum values. (**B**) Heat maps illustrating EA expression in immature myeloid cell subpopulations (HSCs, CD34^+^ myeloblasts, CD117^+^ myeloblasts, and monoblasts) in AML M4/M5 and non M4/M5-AML compared to NBM. *, *p*-value ≤ 0.05.

Significantly fewer Nanog-positive (1.9% vs. 34.0%, *p* = 0.017), SOX2-positive (0.4% vs. 6.1%, *p* = 0.031), and SSEA-3-positive (50.9% vs. 89.7%, *p* = 0.047) HSCs were detected in AML M4/M5 samples compared to NBM. In addition, AML M4/M5 samples contained significantly fewer Nanog-positive CD34^+^ myeloblasts than normal samples (0.5% vs. 11.6%, *p* = 0.017) (Figure 5A). For the remaining cell subpopulations, no significant differences in the percentages of EA-expressing cells were observed between M4/M5 AML and normal samples. The immature myeloid cells in AML without a monocytic component (non-M4/M5 AML) did not express EAs (MMI < 10^1^ for all EAs; Figure 5B).

Reduced expression of OCT3/4, Nanog, and SOX-2 was observed in AML M4/M5 cells compared to normal HSCs (OCT3/4 MMI: 12.1 ± 1.4 vs. 41.5 ± 25.2, *p* = 0.0025; Nanog MMI: 12.6 ± 1.6 vs. 49.9 ± 30.2, *p* = 0.0025; SOX2 MMI: 12 ± 1.4 vs. 43.8 ± 17.8, *p* = 0.0158). In addition, an abnormal increase in SSEA-1 and a reduction in SSEA-3 were observed in AML M4/M5 HSCs compared to their normal counterparts (SSEA-1 MMI: 40.3 ± 13.6 vs. 14.7 ± 0.4, *p* = 0.0194; SSEA-3 MMI: 20.4 ± 6.0 vs. 29.2 ± 6.7, *p* = 0.0303). Unlike the normal myeloblasts, however, CD34^+^ myeloblasts from AML M4/M5 expressed higher levels of SOX2 than those in NBM (MMI SOX2: 38.5 ± 6.4 vs. 11.3 ± 0.6, *p* = 0.0257).

Similar to the HSCs, CD34^+^ and CD117^+^ myeloblasts from AML M4/M5 expressed lower levels of SSEA-3 than their counterparts in NBM (SSEA-3 MMI in CD34^+^ myeloblasts: 14.1 ± 0.8 vs. 18.5 ± 3.3, *p* = 0.0735; SSEA-3 MMI in CD117^+^ myeloblasts: 16.3 ± 1.9 vs. 22.7 ± 4.5 MMI, *p* = 0.0176) as well as increased levels of SSEA-1 (SSEA-1 MMI in CD34^+^ myeloblasts: 15.8 ± 4.3 vs. 13.0 ± 1.1, *p* = 0.2895; SSEA-1 MMI in CD117^+^ myeloblasts: 35.9 ± 7.7 vs. 17.8 ± 0.8, *p* = 0.0638). We also observed reduced expression of SSEA-1 and SSEA-3 in AML M4/M5 monoblasts compared to their normal counterparts (SSEA-1 MM1: 12.8 ± 0.2 vs. 17.7 ± 2.1, *p* = 0.0507; SSEA-3 MMI: 13.6 ± 1.1 vs. 17.5 ± 3.9, *p* = 0.0479).

To conclude, an overall reduction in EA expression was observed in HSCs from AML M4/M5 samples. Moreover, EA expression was absent in non-M4/M5 AML compared to normal HSCs. However, increased SSEA-1 and decreased SSEA-3 expression were observed in HSCs and myeloblasts from AML M4/M5 compared to their normal counterparts. For monoblasts, reduced SSEA-1 and SSEA-3 expression was observed in AML M4/M5 samples compared to normal samples, whereas CD34^+^ myeloblasts from AML M4/M5 samples expressed more SOX2 than their normal counterparts.

### 3.4. Evaluation of β-Catenin, p-p38, STAT3, and p-STAT3 Expression in Immature Myeloid Cells from AML and NBM

Next, we evaluated the cytoplasmic expression of two phosphorylated proteins and two transcription factors frequently involved in leukemic cell-associated SPs.

**Figure 6:**
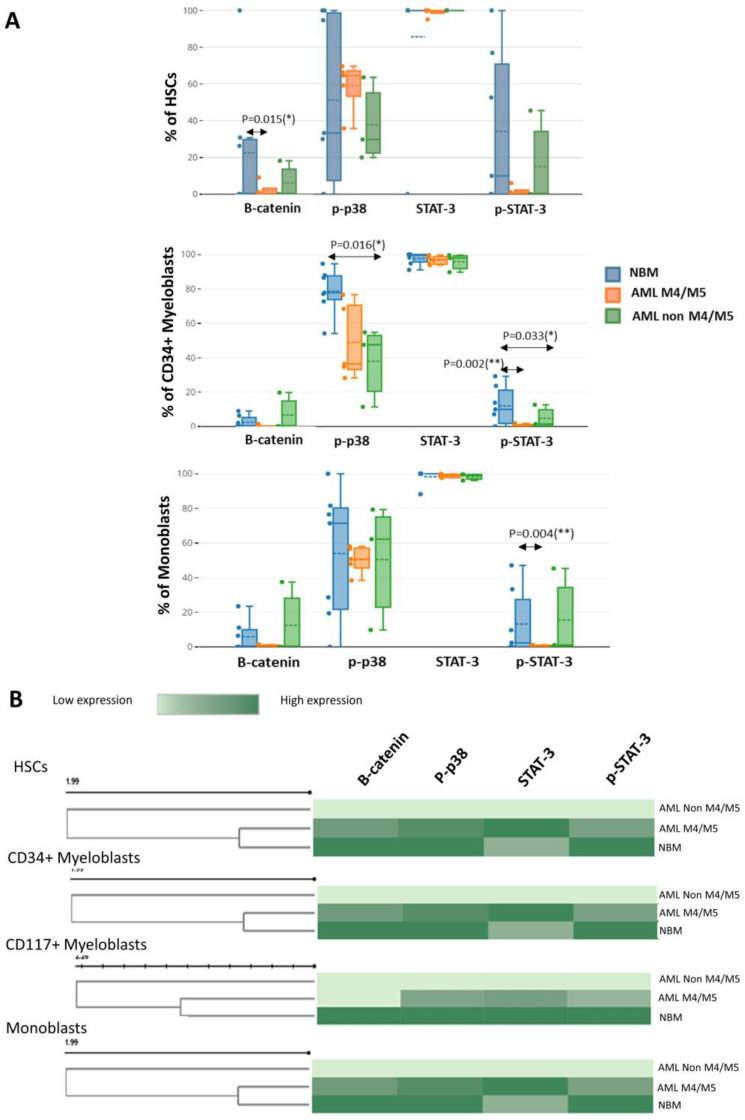
Evaluation of the differences in signaling protein (SP) expression (β-catenin, p-p38, STAT3, and p-STAT3) between acute myeloid leukemia (AML) and normal bone marrow (NBM) by mass cytometry. (**A**) Box plots showing the percentages of SP-positive hematopoietic stem cells (HSCs) and CD34^+^ myeloblasts in AML M4/M5 and non-M4/M5 AML compared to NBM. Median values are indicated by the solid line inside the box, the dashed line indicates the mean value, q3 represents the upper boundary, q1 represents the lower boundary, and the ends of the whiskers indicate the maximum and minimum values. (**B**) Heat maps illustrating SP expression in immature myeloid cell subpopulations (HSCs, CD34^+^ myeloblasts, CD117^+^ myeloblasts, and monoblasts) in AML M4/M5 and non-M4/M5 AML compared to NBM. *, *p* ≤ 0.05; **, *p* ≤ 0.01.

Although myeloid cells constitutively express STAT3 both in normal settings and leukemia, we noticed a smaller number of myeloid cells expressing p-STAT3 in leukemia. Specifically, significant differences in leukemic cells compared to their normal counterparts were observed for CD34^+^ myeloblasts (AML M4/M5 vs. NBM: 1.0% vs. 13.7%; non-M4/M5 AML vs. NBM: 3.1% vs. 13.7%, *p* = 0.0333) and monoblasts (AML M4/M5 vs. normal monoblasts: 1.2% vs. 12.1%, *p* = 0.0043) (Figure 6A).

A reduced percentage of β-catenin-positive HSCs was observed in AML samples compared to control BM (AML M4/M5 vs. NBM: 1.2% vs. 22.2%, *p* = 0.0158; non-M4/M5 AML vs. NBM: 5.3% vs. 22.2%) (Figure 6A).

We also observed a reduced proportion of p-p38-positive CD34^+^ myeloblasts in AML compared to normal settings, but statistical significance was only attained for non-M4/M5 AML (non-M4/M5 AML vs. NBM: 48% vs. 83.6%, *p* = 0.0166; AML M4/M5 vs. NBM: 33.7% vs. 83.6%, *p* = 0.0732) (Figure 6A).

In addition, we also observed differences in the expression levels of these proteins in immature myeloid cells from AML compared to NBM. Overall, we observed reduced β-catenin expression in immature myeloid cells from AML compared to their normal counterparts, but statistical significance was only attained for HSCs (AML M4/M5 vs. NBM MMI: 13.1 vs. 28.0, *p* = 0.0139; non-M4/M5 AML vs. NBM MMI: 14.2 vs. 28.0, *p* = 0.0217). A reduction in p-p38 intensity was also observed in immature cells from AML compared to normal counterparts but did not achieve statistical significance (Figure 6B). Increased STAT3 expression was observed in immature myeloid cells from AML M4/M5 compared to their normal counterparts; statistical significance was attained for HSCs (AML M4/M5 vs. NBM MMI: 205.4 vs. 166.4, *p* = 0.0479). However, constitutive p-STAT3 expression was more strongly reduced in immature AML cells, including HSCs, from all AML settings compared to their normal counterparts (Figure 6B).

### 3.5. Mass Cytometry-Based Evaluation of SPs in Neutrophils from AML and Normal Controls

**Figure 7:**
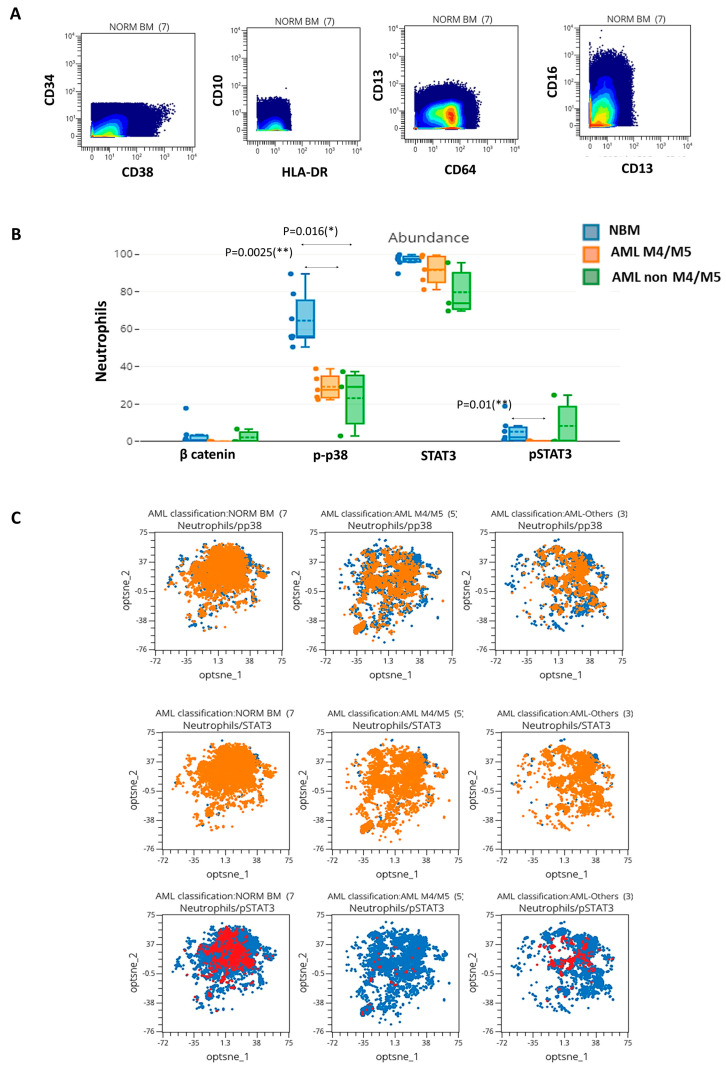
Evaluation of signaling protein (SP) expression in neutrophils by mass cytometry. (**A**) Bivariate dot plots illustrating the relevant immunophenotypic features of neutrophils. (**B**) Box plots showing the percentages of SP-positive (β-catenin, p-p38, STAT3, and p-STAT3) neutrophils in non-M4/M5 AML and AML M4/M5 compared to normal samples. The median value is indicated by the solid line inside the box; the dashed line inside the box indicates the mean value; q3 and q1 represent the upper and lower boundaries, respectively; and the ends of the whiskers indicate the maximum and minimum values. The blue, orange, and green boxes represent normal controls, AML M4/M5, and non-M4/M5 AML, respectively. (**C**) opt-SNE allowing visualization of p-p38, STAT3, and p-STAT3 expression in neutrophils. First row: p-p38 expression overlaid on opt-SNE plots. Orange and blue dots correspond to p-p38-positive and p-p-38-negative neutrophils, respectively. Second row: STAT3 expression overlaid on opt-SNE plots. The orange and blue dots correspond to STAT3-positive and STAT3-negative neutrophils, respectively. Third row: p-STAT3 expression overlaid on opt-SNE plots. The red and blue dots correspond to p-STAT3-positive and p-STAT3-negative neutrophils, respectively. First column: normal controls (NBM); second column: AML M4/M5; third column: other AML (non-M4/M5 AML). *, *p* ≤ 0.05; **, *p* ≤ 0.01.

Next, we analyzed the expression of phosphorylated proteins in mature myeloid cells from AML samples and their normal counterparts. These proteins were only differentially expressed in neutrophils (immunophenotypically characterized in Figure 7A). A significative reduction in the percentages of p-p38 positive neutrophils was detected in AML M4/M5 vs. NBM (26.9% vs. 53.6%, *p* = 0.0025) and non-M4/M5 AML vs. NBM (27.5% vs. 53.6%, *p* = 0.0166) (Figure 7B,C). In addition, we observed a reduction in p-p38 expression in neutrophils from AML settings compared to normal samples (MMI AML M4/M5 vs. NBM: 23.1 vs. 39.6, *p* = 0.0344; MMI non-M4/M5 AML vs. NBM: 20.1 vs. 39.6, *p* = 0.0222). Finally, we observed a significant reduction in the percentages of p-STAT3-positive neutrophils in AML M4/M5 compared to NBM (0.2% vs. 8.3%, *p* = 0.0101) (Figure 7B,C). In conclusion, reduced activation of p-p38 and p-STAT3 was observed in neutrophils in the setting of AML.

## 4. Discussion

AML represents a heterogeneous group of hematological malignancies characterized by the abnormal proliferation of undifferentiated myeloid progenitors [9]. Various studies have reported aberrant expression of key pluripotency transcription factors (OCT4, NANOG, and SOX2) in different types of solid and hematological tumors [4,5,6]. A previous study by our team showed that shRNA-mediated OCT4 downregulation in AML cell lines resulted in differentiation arrest in at least some types of AML and that it also plays a role in cell proliferation [10]. In this context, the objective of the present study was to explore the contribution of OCT3/4 and OCT3/4-regulated EAs in leukemogenesis by mass cytometry. Moreover, we interrogated several SPs (β-catenin, p-p38, STAT3, and p-STAT3), the expression of which is regulated by OCT3/4. These SPs are involved in major hematopoiesis regulatory pathways, such as the Wnt/β-catenin, JAK/STAT, and RAF/MEK/ERK (MAPK) pathways, and are frequently affected by mutations in AML cases.

Although a small number of cases were included, the present study is original and builds upon the few prior studies dedicated to exploring myeloid cell EAs and SPs in AML by mass cytometry. Herein, we designed a 36-antibody panel according to the recommendations of Euroflow [11], allowing us to distinguish among BM cell populations—particularly the myeloid cell populations—and evaluate expression of EAs and EA-regulated SPs. Combinatorial expression of multiple phenotypic markers facilitated the exhaustive exploration and precise identification of 14 BM cell populations in the absence of morphological parameters, among which included cell populations relevant to the diagnosis of AML: HSCs, CD34^+^ myeloblasts, more mature CD117^+^ myeloblasts, monoblasts, and immature monocytic cells. In addition, we adapted labeling and mass cytometry acquisition protocols to BM samples and established a strategy for data analysis.

We observed an overall reduction in EAs in HSCs from AML M4/M5 samples, as well as an absence of EA expression in non-M4/M5 AML samples, compared to normal HSCs. This abnormality might affect cell differentiation. In embryonic cells, quantitative expression of OCT3/4 has been used to define differentiation, dedifferentiation, and self-renewal [12], whereas OCT3/4 inhibition in leukemic cell lines was associated with differentiation arrest [10]. The post-translational modification of OCT3/4 by deubiquitinating enzymes regulates the stability of OCT3/4, and cellular deficiencies in OCT3/4 have been linked to the inhibition of cell differentiation in cancer [13]. OCT3/4 also regulates the expression of other transcription factors such as SOX2 and Nanog, which were also downregulated in AML. These factors influence other cellular programs tightly linked to differentiation and pluripotency (in the case of SOX2 and Nanog) or self-renewal (controlled by Nanog) [14,15].

We identified increased SOX2 expression in CD34^+^ myeloblasts from AML M4/M5. The pathogenic role of SOX2 in AML is underexplored despite the observation that SOX2 may serve as an oncogene in some cancers [16].

Aberrantly increased expression of SSEA-1, which is a marker for mature cells, along with reductions in the expression of SSEA-3, a marker for immature cells, was observed in HSCs and myeloblasts from AML M4/M5. In addition, reduced expression of SSEA-1 and SSEA-3 was observed in AML M4/M5 monoblasts compared to normal monoblasts. Therefore, the incorporation of these two markers into the panels used for AML M4/M5 follow-up could help distinguish between immature leukemic cells and their normal counterparts.

In line with recent studies that used mass cytometry to dissect intratumoral heterogeneity in AML [17,18], we observed differences in signaling phenotypes between leukemic cells and their normal counterparts. More precisely, mass cytometry has been shown to allow the identification of leukemic cells exhibiting primitive signaling (inferred functionally primitive cells [IFPCs]); thus, this method can overcome the inability of conventional flow cytometry techniques to universally distinguish leukemic stem cells across AML patients in the absence of a specific surface phenotype [17]. Moreover, mass cytometry enables identification of differential signaling pathway expression between primitive and mature leukemic subpopulations [17].

We observed in AML samples a lower intracytoplasmic β-catenin expression in immature myeloid cells than their normal counterparts from the NBM.

Morgan R. G. et al. also noticed an underexpression of the cytoplasmic β-catenin in Wnt-unresponsive AML myeloblasts, and this would be the result of the nuclear localization of β-catenin due a disrupted nuclear-cytoplasmic transport [19]. Importantly, nuclear localization of β-catenin is associated with an adverse prognosis in AML [20,21,22].

Therefore, the abnormal β-catenin expression in AML warrants further investigation.

In addition, we observed increased protein expression levels of STAT3 in HSCs and CD34^+^ myeloblasts from AML M4/M5 but reduced expression levels of p-STAT3, compared to their respective normal counterparts. p-STAT3 was also decreased in immature cells from non-M4/M5 AML samples. The STAT3/5 signaling pathway is induced in leukemic blasts by environmental factors such as granulocyte colony-stimulating factor stimulation or interactions with stromal cells. Narayanan et al. showed that AML patients with low environment-induced STAT3 pathway signaling had inferior outcomes compared to patients with a stronger inducible STAT3 response [23].

Reduced p-p38 and p-STAT3 activation was also observed in neutrophils from AML samples compared to normal controls. The p38 MAPK pathway plays critical roles in neutrophil chemotaxis and the regulation of surface receptor expression [24]; phosphorylating and inhibiting the activities of caspase-8 and caspase-3, thereby hindering neutrophil apoptosis [25]; and controlling the chemoattractant-induced degranulation of neutrophils [26]. Normal signaling through the STAT3 pathway is required for granulocyte differentiation and maturation [27]. Constitutively phosphorylated STAT3 is often observed in leukemic cells from karyotypically normal AML cases but less frequently in leukemic cells from AML cases with recurrent genetic abnormalities such as AML t(8;21) [28]. In addition, Levine et al. observed attenuation of the IL-10–p-STAT3 response in mature immune cells in AML and considered this as another distinguishing feature of IFPCs in this context [17]. Thus, the abnormal signaling phenotype indicates that these neutrophils are functionally abnormal, consistent with the view that they are derived from a malignant clone of myeloid precursor cells.

As previously reported by Levine et al., the preliminary results of our study show that the exploration of intracellular signaling in AML BM cells is more informative than antigen profile evaluation. The former approach captures the functional state of diseased cells, thus allowing the precise identification and quantification of leukemic clone(s).

The possible impact of functional signaling analysis in AML, as well as the prognostic impact of abnormal EA, β-catenin, p-p38, and p-STAT3 expression, needs to be examined in future studies. One limitation of our study is its small cohort size; follow-up studies with larger patient cohorts are required to further validate our findings.

A major advantage of mass cytometry is the ability to measure more than 40 cellular parameters from specimens in which sample volume is limited. However, there are also disadvantages. First, cell loss during the staining process, estimated at ∼30%–50% of the initial cell number [29], can occur, particularly due to labeling cells with iridium-containing intercalators. Nevertheless, these intercalators are necessary because they compensate for a lack of light-scattering properties and are required to ensure that all cells are counted. Live-dead dyes such as cisplatin used to determine cell viability also lead to cell loss [30]. Second, a large proportion of cells is lost in the mass cytometer instrument, resulting in <50% of the injected cell sample being analyzed [31].

## 5. Conclusions

Our work provides a framework for using mass cytometry to characterize EAs and monitor cell signaling phenotypes in myeloid cells from AML BM samples. Functional signaling analysis is more informative than antigen profile evaluation in AML due to extensive intra- and inter-patient immunophenotypic heterogeneity. Therefore, this method may also improve genetic diagnostic approaches to risk stratification.

Mass cytometry is an extremely useful tool to explore antigens with low-intensity expression, such as EAs, and to measure multiple intracellular markers such as phosphorylated proteins. However, further optimization of the technology, labeling protocols, and data acquisition approaches for BM samples is required to improve cell recovery and increase detection of rare events such as leukemic stem cells.

## Figures and Tables

**Table 1:** Patient characteristics.

Patient	Age (Y)	Sex	WHO Classification	BM Cytology	WBC × 10^9^/L at Diagnosis	Blast Counts in BM Aspirate Smears at Diagnosis
AML 2	73	F	AML-MRC	AML non M4/M5	1.61	27%
AML 3	72	F	AML with mutated *NPM1*	AML M4/M5	50.53	39%
AML 7	85	F	AML NOS, acute monoblastic leukemia	AML M4/M5	79.04	65%
AML 8	76	F	“Therapy-related” AML	AML M4/M5	26	55%
AML 11	71	M	AML with mutated *NPM1*	AML M4/M5	51.92	66%
AML 12	61	F	“Therapy-related” AML	AML non M4/M5	11.49	82%
AML 14	1	F	AML NOS, AML with maturation	AML non M4/M5	6.52	59.5%
AML 15	52	M	AML-MRC	AML non M4/M5	21.89	27.5%

Abbreviations: F, female; M, male; AML, acute myeloid leukemia; *NPM1*, nucleophosmin gene; NOS, not otherwise specified; MRC, myelodysplasia-related changes; AML M4/M5, acute myeloid leukemia with monocytic component; AML non M4/M5, acute myeloid leukemia without monocytic component; WBC, white blood cell; FAB, French–American–British; BM, bone marrow.

## Data Availability

The data presented in this study are available from the corresponding author upon request.

## Supplementary Materials

The following supporting information can be downloaded at https://www.mdpi.com/article/10.3390/cancers15194707/s1: Figure S1: The manual gating strategy for the identification of cell populations; Figure S2: Representative dot plots for immunophenotypic characterization of immature BM cells; Figure S3: Distribution of the immature myeloid populations in NBM, AML M4/M5, and AML non M4/M5 settings. Table S1: Mass cytometry antibody panel. Table S2: Sample preparation for mass cytometry analysis.
