# Supplementary material for: High-Dimensional Mass Cytometry Analysis of Embryonic Antigens and Their Signaling Pathways in Myeloid Cells from Bone Marrow Aspirates in AML Patients at Diagnosis
Source: Cancers (Basel). 2023 Sep 25;15(19):4707. doi: 10.3390/cancers15194707 (PMC10571794; doi:10.3390/cancers15194707)
Supplement: Supplementary file 1 [file cancers-15-04707-s001.zip › cancers-2548096-supplementary.pdf]

Figure S1: The manual gating strategy for the identification of cell populations.

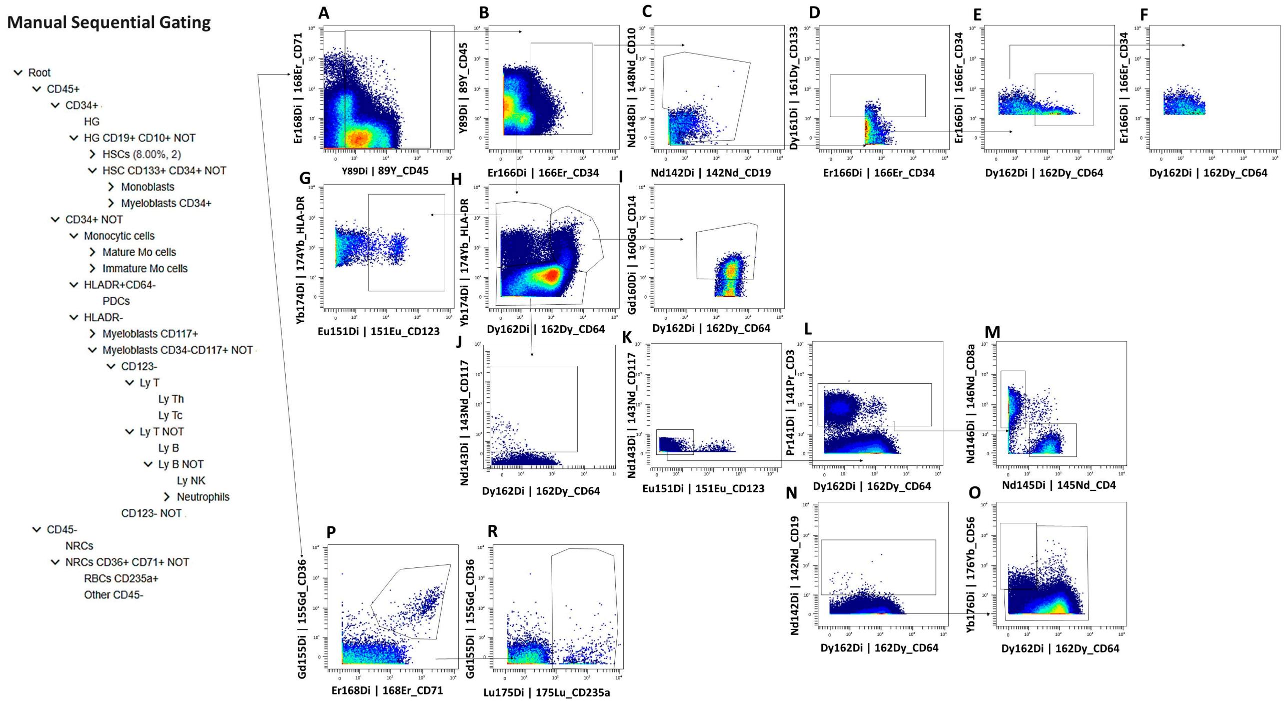

(A) Selection of CD45+ cells. (B) Selection of CD34+ cells. (C) Removal of CD10+CD19+ hematogones (HGs) from the CD34+ cell gate. (D) Selection of CD133+ hematopoietic stem cells (HSCs) within the CD34+ cell gate after HG removal. (E) Selection of CD64+ monoblasts within the CD34+ cell gate after exclusion of CD133+ HSCs and HGs. (F) The remaining CD34+ myeloblasts. (G) Selection of CD123+HLA-DR- basophils from among the CD64+low/-HLA-DR- cells. (H) Selection of CD64+HLA-DR+ monocytic cells and CD64+low/-HLA-DR- neutrophils. (I) Selection of CD14+CD64+ more mature monocytic cells. (J) Selection of CD117+ cells from among CD64+low/-HLA-DR+low/- cells. (K) Selection of CD117-CD123- cells. (L) Selection of CD3+ T-lymphocytes. (M) Selection of CD8+ cytotoxic T-lymphocytes and CD4+ helper T-lymphocytes. (N) Selection of CD19+ B-lymphocytes. (O) Selection of CD56+ lymphocytes. (P) Selection of CD36+CD71+ erythroid cells. (R) Selection of CD235a+ erythrocytes.

Figure S2: Representative dot plots for immunophenotypic characterization of immature BM cells

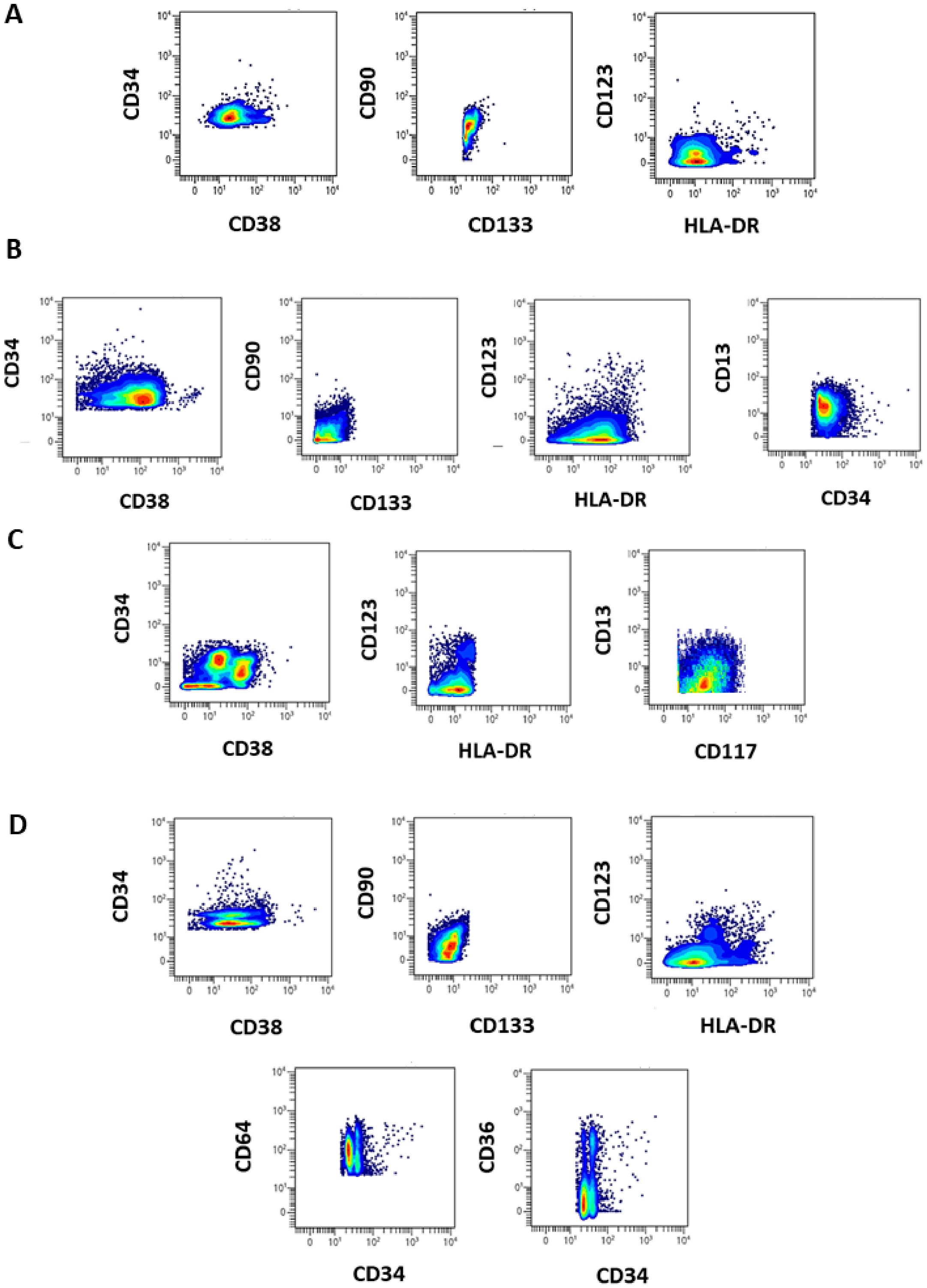

(A) Hematopoietic stem cells (HSCs) CD34+ CD38+^{low} CD90+^{low} CD133+^{low} HLA-DR-/+^{low}; (B) CD34+ Myeloblasts CD34+ CD38+^{low} CD90- CD133- HLA-DR+^{low} CD13+/-; (C) CD117+ Myeloblasts CD34- CD38+ CD123-/+ HLA-DR+^{low}/- CD117+ CD13+/-; (D) Monoblasts CD34+^{low} CD38+ CD90-/+^{low} CD133-/+^{low} HLA-DR+^{low} CD64+ CD36-/+

Figure S3: Distribution of the immature myeloid populations in NBM, AML M4/M5, and AML non M4/M5 settings

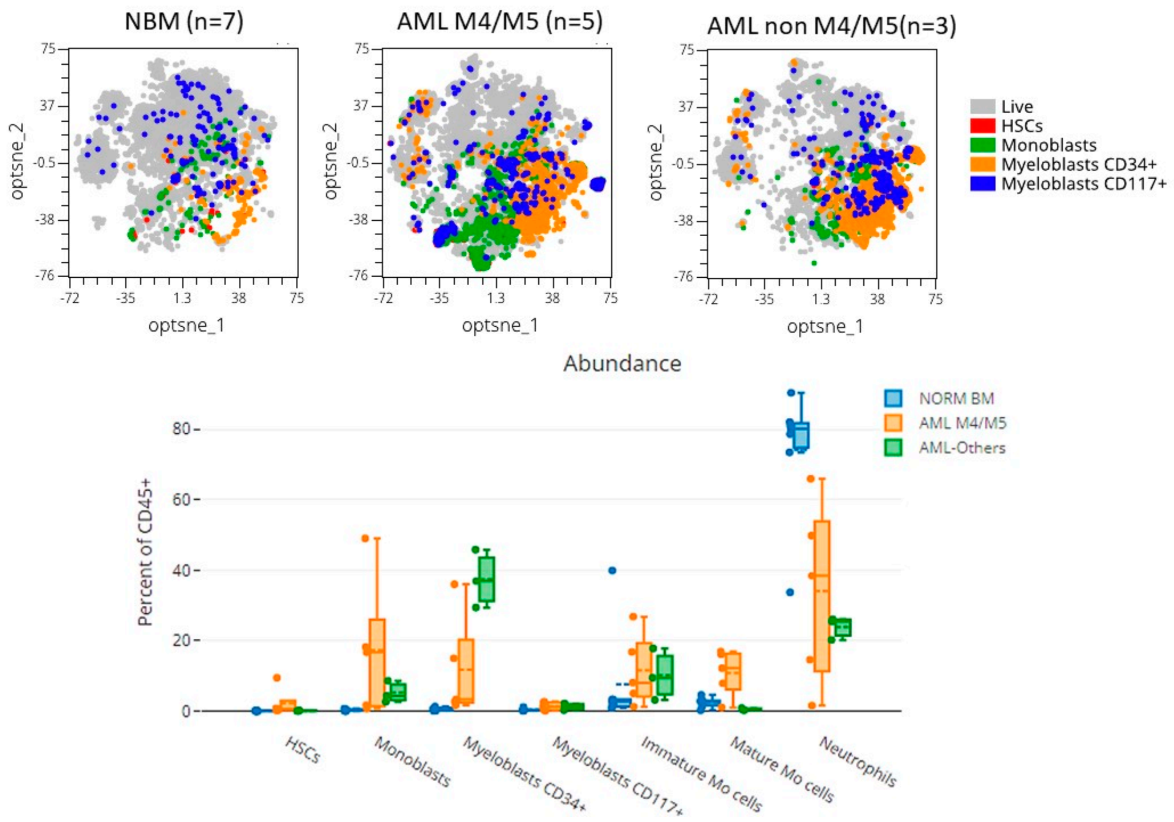

(A) opt-SNE visualization of the distribution of the immature myeloid cells in the bone marrow aspirates from the three groups of cases; (B) Cell population frequencies among CD45-positive leukocytes from normal controls (NBM; blue columns), AML with monocytic component (M4/M5, orange) and AML without monocytic component (non M4/M5, green).

Table S1. Mass cytometry antibody panel.

Label	Target	Clone
089Y	CD45	HI30
141Pr	CD3	UCHT1
142Nd	CD19	HIB19
143Nd	CD117 (c-KIT)	104D2
144Nd	CD15 (SSEA-1)	W6D3
145Nd	CD4	RPA-T4
146Nd	CD8a	RPA-T8
147Sm	β-catenin	D10A8
148Nd	CD10	HI10a
149Sm	SSEA-3	MC-631
150Nd	SOX2	O30-678
151Eu	CD123 (IL-3R)	6H6
152Sm	CD13	WM15
153Eu	CD7	CD7-6B7
154Sm	CD33	P67.6
155Gd	CD36	5-271
156Gd	p-p38 [T180/Y182]	D3F9
158Gd	p-STAT3 [Y705]	4/P-Stat3
159Tb	CD90	5E10
160Gd	CD14	M5E2
161Dy	CD133	AC133
162Dy	CD64	10.1
163Dy	CD105 (endoglin)	43A3
164Dy	CD49F	G0H3
165Ho	OCT3/4	40/Oct-3
166Er	CD34	581
167Er	CD38	HIT2
168Er	CD71 (transferrin receptor)	OKT-9
169Tm	Nanog	N31-355
170Er	CD45RA	HI100
171Yb	CD20	2H7

173Yb	STAT3	124H6
174Yb	HLA-DR	L243
175Lu	CD235ab (glycophorin)	HIR2
176Yb	CD56 (NCAM)	NCAM16.2
209Bi	CD16	3G8

Table S2. Sample Preparation for Mass Cytometry Analysis

Steps #	
0	Perform Bulk Erythrocyte Lysing<br>(https://www.bdbiosciences.com/content/dam/bdb/marketing-documents/Multicolor-Bulk-Erythrocyte-Lysing-Protocol.pdf)<br>Count the cells. If needed, the cells can be kept on Cell wash + FCS (1000 µl cell wash+200 µl FCS)
1	**From Cells of interest**<br>In Eppendorf tubes not autoclaved add around 8 million cells
2	**Viability Staining - Cisplatin**<br>Wash once with 1000 µL MaxPar PBS at RT<br>Add 1000 µL of Cisplatin Solution (CisPlatin 10M 2µl/1000µl in pre warmed medium without FCS) per Eppendorf,<br>Remove Cisplatin Solution (centrifuge cell suspension at 800 x g for 5 minutes).<br>Add 1000-1500µL CSB. Filter the cells. Count the cells. Wash twice with CSB at RT<br>(centrifuge cell suspension at 800 x g for 5 minutes).
3	**Block surface Fc Receptors**<br>Add 50 µL of Fc Block/ sample, mix by pipetting (refer to product data sheet to verify dilution of stock for the Fc block antibody used)<br>Incubate 10 minutes at RT
4	**Fixation**<br>Add 500 µL of Fix I buffer (1X = dilution 1/5)/ sample<br>Incubate 10 minutes at RT<br>Wash with 1000 µL CSB/ sample (centrifuge cell suspension at 800 x g for 5 minutes)
5	**Surface Staining**<br>Add 50 µL of surface antibody mix per sample<br>Incubate 30 minutes at RT<br>Wash 1000 µL CSB/ sample (centrifuge cell suspension at 800 x g for 5 minutes)
6	**Phosphorylated proteins +Intracellular Antigens staining**<br>Resuspend cells in residual volume<br>Add 1 mL of cold 4°C MeOH, mix gently and incubate for 15 minutes on ice<br>Add 500 µL of CSB, centrifuge cell suspension at 800 x g for 5 minutes<br>Wash a second time with 1.5 mL of CSB<br>Resuspend cells in residual volume and add 50 µL of phosphorylated proteins +intracellular antibodies cocktail<br>Gently pipet to mix samples then incubate for 30 minutes at RT<br>Wash with CSB (centrifuge cell suspension at 800 x g for 5 minutes)
7	**Fresh fix**<br>Resuspend cells in 1000 µL 3.2 % PFA solution (200µl PFA + 800µl PBS/ 1 sample)<br>Incubate 10 minutes at RT<br>Centrifuge cell suspension at 800 x g for 5 minutes, discard supernatant
8	**Fixation overnight**<br>Resuspend cell pellet in 500uL **Cytofix + 1/1000 Iridium**

	Incubate overnight at 4°C
**9**	**Freezing**<br>Add 500 µl CSB. Resuspend the cells. Count the cells.<br>Centrifuge cell suspension at 800 x g for 5 minutes, discard supernatant.<br>Add "freezing solution": 135 µl CSB+ 7.5 µl DMSO + 7.5 µl PBS/ sample<br>Resuspend pellet in the "freezing solution"<br>Place tubes at -80°C
**10**	**Cell preparation for acquisition**<br>Thaw sample on ice<br>Add 1000 µl CAS solution. Count the cells<br>Centrifuge cell suspension at 800 x g for 5 minutes, discard supernatant<br>Resuspend cells in CAS solution + 10% EQ beads for a final concentration of 200.000 cells / mL<br>Acquire sample by mass cytometry

FCS, fetal Calf Serum; **PBS**, Phosphate-buffered saline; **RT**, Room Temperature; **CSB**, Cell Staining Buffer; **CAS**, Cell Acquisition Solution; **MeOH**, Methanol; **PFA**, Paraformaldehyde; **DMSO**, Dimethylsulfoxide; **EQ beads**, EQ™ Four Element Calibration Beads
